# Effectiveness of an escape room for undergraduate interprofessional learning: a mixed methods single group pre-post evaluation

**DOI:** 10.1186/s12909-021-02666-z

**Published:** 2021-04-20

**Authors:** Leigh Moore, Narelle Campbell

**Affiliations:** Flinders University, PO Box 41326, Darwin, Casuarina NT 0811 Australia

**Keywords:** Interprofessional, Education, Escape room, Game, Student, Health

## Abstract

**Background:**

Interprofessional practice (IPP) has been shown to contribute to quality service provision and improved health outcomes. This knowledge has led to the integration of interprofessional education into course curricula for many health-care disciplines. Offering interprofessional education in rural areas to students undertaking work integrated learning placements is challenging particularly because of the diversity of students and placement dates combined with the student focus on the assessable placement curriculum.

This research investigated and evaluated the utility of an escape room as an educational modality that facilitates learning whilst providing a supportive and motivating learning environment. Our project focused on the acquisition of interprofessional practice knowledge and experience by a health professional student cohort.

**Methods:**

This study used the novel intervention of an escape room combined with an interactive teaching session to test student engagement and learning about interprofessional practice and teamwork. The research used a mixed methods single group pre-post design.

**Results:**

Fifty students (78% female) from seventeen universities and seven professions participated in teams of three to six members. Most participants (66%) had not previously completed an escape room. The results showed that the intervention provided effective and engaging learning and was intrinsically appealing to students despite its non-assessable nature. Individual student reflection on their participation showed developing insight into the critical importance of clear communication and intentional team member collaboration in the provision of effective interprofessional practice.

**Conclusions:**

The escape room intervention added value to the placement curriculum and proved flexible for a heterogeneous student cohort.

**Supplementary Information:**

The online version contains supplementary material available at 10.1186/s12909-021-02666-z.

## Background

Collaborative or interprofessional practice (IPP) involves multiple health workers from different professional backgrounds working together with patients, their families, carers and communities to deliver health care [1, 2]. IPP has been shown to contribute to improved patient outcomes and quality service provision [1]. With interprofessional competencies being a core component of many health professions, both in Australia and internationally, participating in interprofessional learning opportunities is an important foundation for students in developing best practice care [3–5].

In Australia, regional, rural and remote areas have a shortage of health workforce, in part because university-based health professional training programs tend to be located in major cities and rural training exposure (e.g. placements) tend to be of short duration [6]. One strategy underpinned by government policy to address this shortage, has been to ensure health professional students can experience quality placement education in rural and remote areas [7]. Our region, the entire Northern Territory, is sparsely populated with about 250,000 people spread over 1.3million square kilometres [8]. About 30% of the population are Aboriginal people and most of these live in very remote areas, some of the most remote locations in Australia [9, 10]. In this region, our organisation annually supports more than 500 hundred students, from over 20 health professions and up to 30 universities, who are undertaking workplace learning. These students are learning their profession in our acute and primary health services, schools, and community-based settings in urban, rural and remote locations.

Recognising the importance of interprofessional practice, our organisation is working to embed interprofessional learning into placement offerings for all students [5]. This is somewhat challenging due to the heterogeneity of our student body previously described and because the students typically are focused on meeting their home university placement outcomes. Any additional educational activities we provide need to value-add to the placement curriculum and be sufficiently flexible to adapt to the diversity of the cohort. As these additional activities are non-assessable, they also need to be engaging, effective and able to be used with groups of students who may spend no more than a few hours together. To meet these divergent requirements, we undertook a series of research projects on the utility of escape rooms in delivering interprofessional education.

Escape rooms are a relatively recent international entertainment phenomenon and are immersive “team-based games where players discover clues, solve puzzles, and accomplish tasks in one or more rooms in order to accomplish a specific goal … in a limited amount of time” [11]. Escape rooms may include a ‘game master’ who observes the progress of participants through the room and is available to give hints if required to ensure participants do not become unduly stuck on any puzzle.

The literature on escape rooms as educational environments, and the benefits of gamification where game elements are used in health education [12] is growing. In particular, evidence on their applicability to tertiary healthcare education remains limited. At commencement of our project, the literature available on health themed escape rooms was mostly from the United States of America [13–25] with two studies from Spain [26, 27] and two from Canada [28, 29]. The literature showed significant heterogeneity in purpose, room design and evaluation methods making direct comparison difficult. Of the available studies, none were Australian and only two [22, 24] were used interprofessionally. Although with sometimes small participant numbers, quasi-experimental design or being descriptive in nature, and describing heterogeneous participants and aims, the papers indicated positive learning outcomes, student appeal, and provided valuable suggestions on puzzle development and managing challenges. A more recent systematic review on escape rooms as a learning environment indicated that educational ones ‘can provide an enjoyable experience that immerses students as active participants’ and provide ‘opportunity to engage in an activity that rewards teamwork, creativity, decision-making, leadership, communication, and critical thinking’ [30]. This review did not specifically focus on healthcare or interprofessional applicability but indicated the potential of incorporating escape rooms as a novel, engaging way of delivering education.

As a result of the body of available evidence and our requirements to provide an educational session that both demonstrated team functioning and provided a social activity for students, a purpose-designed educational session incorporating an escape room was developed. This was to be used as a novel mechanism for interprofessional learning for placement students in our region and a means of contributing to the growing body of literature around escape rooms. With the assistance of a professional escape room designer, an escape room with a scenario which authentically highlighted the unique aspects of working as a health professional in our isolated tropical context, Nana’s Nightmare, was designed and piloted [31]. Following the pilot, the escape room was refined, and an educational session developed that addressed the needs of health professional students to learn more about healthcare systems, other healthcare professions, interprofessional practice and teamwork.

This paper describes our research evaluating the effectiveness of using the format of the escape room to deliver an educational activity. The overall research question is whether an educational session incorporating an escape room can facilitate the acquisition of interprofessional practice knowledge and competencies in health professional students. Specific evaluation objectives were:

1. To investigate the utility of an escape room coupled with a debriefing workshop as an effective and engaging interprofessional learning activity
2. To evaluate the impact of the escape room on participant knowledge about interprofessional practice and teamwork
3. To evaluate the impact of the escape room through participant reflection on their personal contributions to the team

## Methods

The research used a mixed methods single group pre-post design [32] to evaluate the effectiveness of the escape room session..

### Participants

Participants, health professional students, were recruited from the University database using email, via social media, posters in student common areas and workplaces, and indirectly through clinical supervisors and course coordinators. Snowball recruitment [33] occurred via recommendations from previous participants and supervisors. Up to six students were able to book into each session. All teams comprised at least two disciplines/professions. The recruitment advertising and participant information indicated that participants would contribute to research knowledge about the educational benefits of escape rooms while also learning about interprofessional practice and teamwork.

### Intervention

The intervention was a session comprised of two components. It commenced with the escape room and was followed by an educational workshop focused on teamwork and interprofessional practice. Development and delivery of our portable, educational escape room for this project was completed with a budget of $3000 AUD. This included initial consultation fees for a professional escape room designer. The ‘room’ consisted of nine health-themed puzzles (for example, one lock could be opened based on Nana’s blood pressure reading), props (including pill boxes, a medication chart, blood pressure monitor, an inflatable mannequin dressed as the key character), and lesson materials that could be packed into a large suitcase and set up in most lecture or meeting-type rooms.

The escape room started with an announcement to participants that the region was experiencing a cyclone and as a team of health professionals they had been requested to assist a residential aged care facility. Participants completed a locum staff registration form and then as a team undertook a check of the facility to ensure that all residents were safe. The team was provided with an emergency kit to assist residents as needed. During their room check, the team ‘discovered’ a resident, Nana, fallen out of bed and unconscious. By solving a series of puzzles that highlighted various health professional roles and Nana’s healthcare issues, the final puzzle required the team to call an ambulance for Nana and thus ‘escape the room’. After their escape, the team worked together to develop a health care plan for Nana and then participated in the workshop component. Table 1 details the components of the session, including the purpose of each component, the role of researchers, and the data collected at each point.

**Table 1:** The Escape room intervention detailing session components and their purpose, the role of the researchers at each point and the research data collection framework

Session component	Purpose of component (Role of researchers)	Data collected
Welcome and formal consent (5 min)	Ethical conduct of project (Facilitator)	Consent
Escape room scenario introduction (10 min)	Escape room explanation and commencement Data collection (Actor introducing scenario)	Individual participant demographic details and pre-intervention definition of interprofessional practice (via ‘locum staff registration form’)
Escape room participation (45 min)	Simulate interprofessional practice including health professional roles and teamwork (Observer and game master)	
Comfort break and health care plan development (15 min)	Simulate interprofessional practice and teamwork.	Health care plan result was incorporated into educational session
Educational session (60 min)	Teamwork, interprofessional practice reflection and interactive learning (Facilitator)	Team effectiveness questionnaire results
Evaluation of learning (15 min)	Data collection (Facilitator)	Post-intervention definition of interprofessional practice Content knowledge quiz Reflective evaluation Self-evaluation of learning against the learning objectives^1^

^1^See additional file 1

The simulated, locum staff registration form completed by participants at the commencement of the escape room component collected participant demographic characteristics and their individual pre-intervention definition of interprofessional practice. The educational session was facilitated by one or two of the researchers and included didactic content and group-work (based on information obtained during the escape room) to present, or elicit:

- the characteristics of an effective team
- the definition, features and benefits of interprofessional practice
- the barriers to, and enablers of, interprofessional practice

During the educational session participants individually assessed the team’s effectiveness on each of 11 team characteristics using an adaptation of Sharif and Nahas’ questionnaire [34–36]. The individual ratings were then collated and fed back to participants as the overall team ratings for each of the attributes using methodology described by Sharif and Nahas [35]. The team results then framed discussion in the educational session. At the end of the session, participants were invited to revise their definition of interprofessional practice and completed a content knowledge quiz and a reflective evaluation. Finally, participants self-rated their pre and post session knowledge for each of the six learning objectives. (1 = low, 5 = excellent). The form used to gather this data is included as additional file 1.

### Analysis

Quantitative data was analysed descriptively and paired-samples t-tests used to compare pre and post session self-rated changes in knowledge. Qualitative data was synthesised using an inductive, descriptive, thematic analysis method based on processes described by Braun and Clarke [37, 38]. Codes were derived from participant written answers and comments, by one researcher. These codes were reviewed by a second researcher, then jointly moderated to assign ambiguous data, and negotiate points of disagreement and develop themes. Prevalence of themes were noted and then data reviewed again to investigate whether individual or outlier comments were able to provide information for improvement of future session delivery.

## Results

### Participants

The session was run eleven times with a total of fifty participants. All participants completed all parts of the session and evaluation. The number of participants in each session ranged from three to six. Most (98%) of participants were aged 19–35 years (mean = 25.5). There were more females (78%) than males (22%). Participants were enrolled in seventeen universities, both Australian and international, with the most students from our own university; and came from years 2–6 of courses from seven different disciplines (Table 2). Most participants (*n* = 33, 66%) had not previously participated in an escape room of educational or entertainment design, some (*n* = 12, 24%) had participated in one room, and one had participated in ten.

**Table 2:** Participants by age and discipline

discipline	gender		age (years)				total sample n (%)
	F	M	< 21	21–25	26–30	31+	
Medical Imaging	19	3	*7*	*13*	*1*	*1*	*22 (44)*
Medicine	4	5		*5*	*2*	*2*	*9 (18)*
Nursing	7	1		*2*	*5*	*1*	*8 (16)*
Occupational Therapy	4		*1*	*2*	*1*		*4 (8)*
Pharmacy	3	1	*3*	*1*			*4 (8)*
Speech Pathology	2		*2*				*2 (4)*
Optometry		1		*1*			*1 (2)*
total sample n (%)	39 (78)	11 (22)	*13 (26)*	*24 (48)*	*9 (18)*	*4 (8)*	50 (100)

### Utility as an effective and engaging interprofessional learning activity

Escape room design is highly variable. The number and difficulty of puzzles, the level of participant immersion and inclusion of electronics and/or proprietary props will be determined by many factors including budget, space, audience, portability and learning objectives. This flexibility allowed us to adapt a product that well suited our requirements.

Participant satisfaction with the session had a mean rating of 9.06 out of ten. Most participants commented that the learning was both engaging and entertaining: ‘super fun and immersive’ (P5). When asked to rank a range of learning formats (live lecture, online lecture, reading the literature, tutorial, escape room session), 90% of participants ranked the escape room session as their preferred format to learn about teamwork and interprofessional practice.

Analysis of participant comments on the reason for their rating showed two main themes: the educational value (48% of participants) and the learning novelty (over 50% of participants). Concerning educational value, participants commented on their knowledge acquisition: *‘*great chance to … learn more about interdisciplinary teamwork and … how I can improve as an effective team player’ (P29). ‘it was very educational’ (P43), ‘have gained insight into the need for IPP and will strive to implement to (sic) future care’ (P2), ‘I learnt a lot especially about interprofessional patient care’ (P35). They also mentioned the authentic nature of the scenario, ‘very hands on/visual, allows teamwork to solve realistic puzzles, very close to real life situations’ (P28), ‘liked the health/medical content’ (P13*).*

Over 50% of participants mentioned the novel nature of the learning in the escape room. They used words such as fun, interesting, enjoyable, entertaining and engaging and specifically valued the organised interactive challenge that the escape room format provided. ‘I enjoyed how the escape room was an entertaining experience and how the debrief session was able to link the activity to interprofessional practice. It made the concept much easier to understand’ (P1).

Three members of one team who were unsuccessful with ‘escaping’ the room rated the value of the experience 5 or less. One criticism was that ‘There didn’t seem to be a clear start with so many clues to work through. It was too manic to learn’ (P4). Interestingly, one member of this team provided two ratings indicating they differentiated between success in the game: ‘a one for ‘not making it out’, and the interactive game format: [a nine for] ‘presenting IPP in a fun interesting engaging manner’ (P48).

### Knowledge of interprofessional practice and teamwork

Participants were asked to recall as many as possible of the eleven characteristics of an effective team which were presented to them during the 60-min educational session. Most participants recalled at least five of these characteristics. The top four characteristics recalled were ‘Communication’ (88%), ‘Clear purpose’ (54%), ‘Appropriate Culture’ (52%) and ‘Suitable leadership’ (52%). ‘Distinct roles’ were recalled by a third (32%) of participants. The least recalled characteristics were ‘Cohesion, trust and social relationship’ (22%), Commitment (14%), Decision-making and Conflict management (14%), ‘Relevant members’ (12%), flexibility (12%) and ‘Adequate resources’ (8%).

Participant pre-session definitions of interprofessional practice ranged from ‘I don’t know’ (P20), through to ‘Interprofessional practice refers to using a multi-disciplinary approach to achieve the best outcome. In allied health this could refer to best patient care and outcome’ (P15). After the session, participants reflected on their original definitions and changed them if desired to reflect their newly acquired knowledge. Thirty-seven (74%) of participants adapted their definition. Overall the changes made indicated the participants had learned of the difference between *inter*professional practice and *multi-*professional practice. Most frequently, participant’s post-session definitions included the concept of ‘working together to achieve a common goal for patient care’.

Paired-samples t-test showed a statistically significant increase in knowledge for all five learning objectives. This is shown in Table 3.

**Table 3:** Pre-post changes in knowledge scores for each escape room learning objective using a Likert scale from 1 to 5

Leaning objective	Mean difference (pre – post)	t (49)	***P*** value
The characteristics of an effective team	1.26	12.83	< 0.001
The contributions of individuals to a team’s effectiveness	1.08	10.16	< 0.001
Definition of interprofessional practice	1.74	12.48	< 0.001
The benefits of interprofessional practice	1.64	12.31	< 0.001
The barriers and enablers of interprofessional practice	1.89	13.15	< 0.001
The role of individual professions in an interprofessional team to improve patient care	1.26	10.45	< 0.001

Analysis of the free-text responses demonstrated participant’s increased understanding of interprofessional practice as a result of the session. The most frequently mentioned learning was the importance of different professions and skills contributing to a team (52%). Other frequently reported learnings were the characteristics of effective teams (28%), the barriers, enablers and benefits of interprofessional practice (26%) and the difference between interprofessional and multidisciplinary practice (22%).

### Participant reflection on contribution to the team

Participants reflected on their individual contributions as a team member during the escape room component. Secondarily, they reflected on whether the workshop component had triggered ideas to increase their effectiveness as an interprofessional team member. There were three main themes in the reflections.

Improving communication (57%) was the most common theme. Participants noted that better communication required listening and recognising the ideas of others: ‘(be) more patient with other’s ideas. Sometimes if I thought someone wasn’t on the right track, they did actually end up being correct’ (P37). Improved communication also required increased discussion and a willingness to contribute your ideas and plans ‘I would make more of an effort to speak out and think out loud so other members of the team could feed off from my ideas’ (P22).

The second theme related to taking a pro-active role (41%) in team relationships and leadership. Contributing more actively is illustrated by the participant who said they would: ‘try to play a more active role in the team’ (P45). Taking a leadership role encompassed understanding individual strengths and contributions as exemplified by: ‘have more clear roles and designated leadership possibly. In terms of my contribution perhaps do more to ensure everyone continues to work collaboratively’ (P24).

The other common theme in responses (35%) focused on planning, increased organisation as a team and working together to solve problems: ‘stop and discuss what our plan is as a team and delegate roles and plan of attack, to avoid confusion and be time efficient’ (P4). This theme involved a recognition that teamwork required intentional effort and time invested by team members in order to get the best outcome: ‘define roles from the beginning’ (P10).

## Discussion

The results appear to support the use of an escape room to engage a cohort of multidisciplinary, multi-university health professional students in learning about interprofessional practice and teamwork. High ratings of the session, combined with participant preference for the escape room over more traditional learning modes such as lectures, aligns with other evidence [30] that escape rooms are engaging. Participation in the escape room was voluntary, so it could be argued that the students had a prior preference for game formats, however, other escape rooms with an educational focus have also been rated highly. For example Cain [14] reported that 89% of participants in a blended environment escape room, enjoyed that over a typical classroom experience. Further evidence of high enjoyment from our study was that word of mouth advertising required us to schedule additional sessions.

Our result of lower enjoyment ratings from participants who failed to ‘escape’ also aligns with the literature. Hermanns et al. [16], who had no participants ‘escaping the room’, reported high participant dissatisfaction, with 25.2% of participants responding negatively or neutrally to whether they felt the escape room was a valuable learning.

The literature shows that learners value authentic over theoretical approaches to learning, and that appropriately contextualised learning supports optimal outcomes in interprofessional education [1]. The gamification of learning in our escape room, with a time limit and a health-themed scenario, was intended to simulate authentic work situations as much as possible. Our findings, that learners linked the escape room scenario with real life, is supported by Brown et al. [13] whose participants agreed that the competitive aspect reflected real-life nursing situations and Jambhekar et al. [17] whose radiology residents agreed that the activity reflected future responsibilities such as multi-tasking, communication and working under stress and time pressure.

It has been argued that game-based learning impacts cognitive development, motivation, and decision making, because it triggers emotion [39]. Heightened emotional states including the sense of achievement from solving the escape room might therefore aid learners to gain new knowledge and perspectives. Statistically significant increases in participant knowledge was shown for all six learning objectives in our escape room. Other studies have shown similar changes in information retention [15, 26, 40], improved clinical practice [21], and motivation to learn [40]. Interestingly one study that used pre- and post-assessments to evaluate learning reported a decline, although participants self-reported improved learning and practice [20].

This study demonstrated increased knowledge about interprofessional practice through the pre-post definitions provided by participants, and about teamwork and health professional roles by participant recall of team characteristics. This result combined with the participant reflection regarding their personal practices in the escape room team was interesting because it highlighted the new insights gained and the positive changes they would consider when contributing in future teams. Most participants recalled ‘communication’ as an important characteristic of teams and additionally highlighted the importance of improving communication to achieve better team outcomes. Our scenario encouraged communication between participants in the team and required participants to actively contribute to collaborative decision making to solve the problem. Interprofessional practice in healthcare provision requires collaboration and communication [1, 4, 41] because of the clear link with improved health outcomes [1, 5]. In a context where many patients speak languages other than English, ensuring that health professional students prioritise best practice in communication skills is critical [42].

The importance of more actively contributing, stepping up to leadership, is an attribute observed in well-functioning teams [35] that was also identified in the reflections by our participants. Our escape room was a challenging situation given that most of the teams comprised individuals not well known to each other. This mimics many healthcare teams where rosters and staff turnover [43, 44] require individuals to work as a team despite a lack of familiarity with each other. However, what the findings also demonstrate is that the escape room without the workshop component, may not have been a sufficient prompt for students to reflect on their own performance. Intentionally providing space and reflective prompts in the workshop was an important enabler that capitalised on the novelty of the escape room and the applied learning in the workshop. The prompts encouraged participants to focus on their leadership (or lack thereof), their communication and their skills and knowledge, all of which are important contributors to safety and quality in healthcare and indicators of interprofessional capabilities [45].

## Limitations

Our study, while comprising a reasonable size sample, was limited by the size of each professional group in the sample which was not reflective of the health workforce profile in our region. In addition, having the knowledge gain measured immediately post-workshop does not indicate the effectiveness of long-term learning. Longer term retention and application of knowledge could be a useful next step in our body of research on escape room learning. In addition, it is acknowledged that participant insights gained from the escape room experience may not directly transfer into workplace team practices, however our results suggest that the experience was beneficial for learning.

## Conclusions

As a standalone session, the escape room format with a post-game debrief and educational workshop appears to promote learning and reflective insights in a diverse range of student professionals. As an innately fun and social activity, it offered a motivating and novel learning environment compared with more traditional methods. Participants were able to participate at their own level of comfort and knowledge and were then scaffolded to reflect on and apply their experience to better understand interprofessional practice and effective team functioning. The benefit of offering the educational activity in this format was that it added value to the placement curriculum, was sufficiently flexible for a heterogeneous student cohort and was intrinsically engaging despite its non-assessable nature. By providing a setting for social learning and motivating students to learn, escape rooms can provide a useful adjunct to traditional classroom learning methods.

## Supplementary Information

**Additional file 1.**

## Data Availability

The datasets used and/or analysed during the current study are available from the corresponding author on reasonable request.

## Abbreviations

IPP: Interprofessional practice
