# Supplementary material for: Effectiveness of an escape room for undergraduate interprofessional learning: a mixed methods single group pre-post evaluation
Source: BMC Med Educ. 2021 Apr 20;21:220. doi: 10.1186/s12909-021-02666-z (PMC8056636; doi:10.1186/s12909-021-02666-z)
Supplement: Supplementary file 1 — Additional file 1. [file 12909_2021_2666_MOESM1_ESM.pdf]

1. List as many characteristics of effective teams as you can remember

2. Thinking about what you now know, please turn the page over and review and edit your 'Agency Screening Tool' definition of interprofessional practice.

3. In the table below, please document the **number** that describes your level of knowledge **BEFORE** participating in this session and your level of knowledge **NOW,** after participating in this session.

**1** = Low	**2** = Fair	**3** = Good	**4** = Very Good	**5** = Excellent

**Knowledge about:**	BEFORE	NOW
a. The characteristics of an effective team		
b. The contributions of individuals to a team's effectiveness		
c. Definition of interprofessional practice		
d. The benefits of interprofessional practice		
e. The barriers and enablers of interprofessional practice		
f. The role of individual professions in an interprofessional team to improve patient care		

4. Thinking about statement (f) above, please give two examples of something you learned today.

5. If you could 'do' the escape room and debrief session again, what, if anything, would you change about **your contribution** to the team?

6. Now that you have experienced an educational escape room, rank the following formats according to your preference for learning about interprofessional practice and teamwork. (1=most preferred)

Escape room session	Live lecture	Online lecture	Reading the literature	Tutorial

7. Out of a max score of 10 (1=poor), how would you rate today's session? Why?
